# Comparative analysis of microbial community structure between healthy and *Aeromonas veronii*-infected Yangtze finless porpoise

**DOI:** 10.1186/s12934-020-01383-4

**Published:** 2020-06-05

**Authors:** Zhigang Liu, Aoyun Li, Yaping Wang, Mudassar Iqbal, Aifang Zheng, Mengmeng Zhao, Zhongkai Li, Nuo Wang, Chao Wu, Daoping Yu

**Affiliations:** 1 College of Life Science, Anqing Normal University, Anqing, 246011 China; 2 Research Center of Aquatic Organism Conservation and Water Ecosystem Restoration in Anhui Province, Anqing Normal University, Anqing, 246011 China; 3 College of Veterinary Medicine, Huazhong Agricultural University, Wuhan, 430070 China; 4 University College of Veterinary & Animal Sciences, The Islamia University of Bahawalpur, Bahawalpur, 63100 Pakistan

**Keywords:** Yangtze finless porpoise, *Aeromonas veronii*, Gut microbiota, High-throughput sequencing

## Abstract

**Background:**

The gut microbiota is a complex ecosystem, which is essential for the metabolism, health and immunity of host. Many diseases have been shown to be closely related to the alteration of intestinal flora. *Aeromonas veronii* as a conditioned pathogen can cause disease in Yangtze finless porpoise through intestinal infections. However, it is not clear whether the disease caused by *Aeromonas veronii* is related to changes of intestinal flora. In the current study, the diversity and composition of gut microbiota in the healthy and *Aeromonas veronii*-infected Yangtze finless porpoise were evaluated by high-throughput sequencing to further investigate the potential association between intestinal flora alteration and pathogen invasion.

**Results:**

A total of 127,3276 high-quality sequences were achieved and 2465 operational taxonomic units (OTUs) were in common among all samples. The results of alpha diversity showed that there was no obvious difference in richness and diversity between healthy and *Aeromonas veronii*-infected Yangtze finless porpoise. *Firmicutes*, *Bacteroidetes* and *Proteobacteria* were the most dominant phyla in all samples. In addition, the healthy Yangtze finless porpoise exhibited higher abundance of *Firmicutes* and *Fusobacteria* than *Aeromonas veronii*-infected Yangtze finless porpoise, while, the level of *Proteobacteria* was decreased. At the genus level, *Paeniclostridium* and *Paraclostridium* were the predominant bacteria genera in the CK (healthy Yangtze finless porpoise) group. In the DIS (*Aeromonas veronii*-infected Yangtze finless porpoise) group, *Lactobacillus* and *unidentified_Enterobacteriaceae* were the dominant bacteria genera and the proportion of *Paeniclostridium*, *Paraclostridium*, *Terrisporobacter*, *Cetobacterium*, *Candidatus Arthromitus*, *Terrabacter* and *Dechloromonas* were reduced.

**Conclusions:**

In conclusion, our results showed that *Aeromonas veronii* infection can alter the gut microbiota of the Yangtze finless porpoise by affecting the number of harmful bacteria and beneficial bacteria.

## Introduction

The Yangtze finless porpoise (*Neophocaena phocaenoides asiaeorientalis*) is a rare species that mainly lives in the Yangtze River basin, Dongting lake and Poyang lake in China. Furthermore, the Yangtze finless porpoise is the only freshwater population of porpoises in the world. However, the survival of Yangtze finless porpoise has suffered serious threats due to decline in water quality and overfishing over the last several decades. According to statistics, the number of Yangtze finless porpoise is gradually declining and less than 2000 are remaining [1, 2]. The Yangtze finless porpoise has been listed as an endangered species by the International Union for Conservation of Nature (IUCN) since 2013. Multiple protection measures including captive breeding, in situ and ex situ conservation have been applied to prevent the continuous reduction of this unique porpoise since the end of the last century. At present, two semi-natural and seven natural reserves have been built.

*Aeromonas* spp., as one of the main pathogens of aquatic animals poses a huge threat to the health of aquatic animals [3, 4]. Liu et al. reported that *Aeromonas veronii* can cause the skin necrosis, visceral hemorrhage and even death of Yangtze finless porpoise [5]. Not only that, *Aeromonas veronii* can cause a variety of diseases in terrestrial animals and humans, such as dysentery, sepsis and necrotizing fasciitis [6]. Most of the studies suggest that *Aeromonas veronii* is an opportunistic pathogen that regulates the expression of virulence factors according to the surrounding environment [7, 8]. The latest research shows that the infection of *Aeromonas veronii* may interact with other bacteria and its pathogenicity may be related to the intestinal flora [9].

The intestinal bacterial community consists of a vast number of different microorganisms including commensals, pathogens and some conditioned pathogens [10, 11]. The intestinal flora plays a vital role in growth, metabolism and immunity of the host [12–14]. In addition, intestinal microorganisms can inhibit the proliferation of pathogenic bacteria in the host by competing with the pathogens for nutrients and adhesion sites [15, 16]. Meanwhile, some gut microbes can also produce several metabolites with bacteriostatic effects to prevent the reproduction of pathogenic bacteria [17]. The stability of intestinal flora is a prerequisite for the intestine to play a mechanical and immune barrier against the invasion of pathogenic microorganisms. However, the stability of intestinal flora can be affected by many intrinsic and extrinsic factors, including temperature, environment, antibiotic, and host phenotypes [18]. Previous studies have shown that intestinal flora alteration is closely related to many diseases including diarrhea, rheumatoid arthritis, diabetes and obesity [19–21]. More importantly, intestinal flora imbalance can lead to some conditional pathogens which may show strong pathogenicity [22, 23]. Previous studies have reported differences in intestinal microbes between healthy and *Aeromonas veronii*-infected grass carp [24]. However, still less is known about the intestinal flora structure in healthy and *Aeromonas veronii*-infected Yangtze finless porpoise. Therefore, the objective of the current study was to analyze the microbial diversity of healthy and *Aeromonas veronii*-infected Yangtze finless porpoise by high-throughput sequencing.

## Materials and methods

### Animals and sample collection

Yangtze finless porpoises used in the present study are raised in Anqing Nature Reserve (Anqing, China). The experimental animals (approximately 3 years old, and four males and four females in each group) were selected in the same water area (Table 1). The clinical symptoms of the diseased Yangtze finless porpoise were depression, conjunctival hemorrhage and skin necrosis. Clinical observation, histopathological examination, bacterial isolation and identification, PCR amplification and gene sequence alignment were used to evaluate etiological agent and ultimately determined that the disease in Yangtze finless porpoises was due to *Aeromonas veronii* infection. The relevant research about disease assessment has been published in the Diseases of Aquatic Organisms [5]. Moreover, the Yangtze finless porpoises in the control group were declared healthy after being examined by the professional veterinarian.

**Table 1:** The information of the Yangtze finless porpoise

Sample	Gender	Body length (cm)	Body weight (kg)
CK1.1	Male	152	54.6
CK1.2	Male	138	49.5
CK1.3	Male	151	58.6
CK1.4	Male	132	46.7
CK1.5	Female	124	42.3
CK1.6	Female	149	52.8
CK1.7	Female	128	45.5
CK1.8	Female	145	48.6
DIS1.1	Male	137	44.2
DIS1.2	Male	133	43.5
DIS1.3	Male	141	45.2
DIS1.4	Male	142	46.3
DIS1.5	Female	131	42.2
DIS1.6	Female	137	42.5
DIS1.7	Female	150	50.4
DIS1.8	Female	128	44.5

CK and DIS indicate the healthy and *Aeromonas veronii*-infected Yangtze finless porpoise, respectively

The medical infusion tube (cut the both ends of the medical infusion tube by scissor) dipped in a small amount of petroleum jelly was slowly and rotationally inserted into the anus 10–15 cm of the Yangtze finless porpoise to obtain fecal samples. In this study, a total of 16 fresh fecal samples were collected from sixteen Yangtze finless porpoise (healthy Yangtze finless porpoise: CK1, CK2, CK3, CK4, CK5,CK6, CK7, CK8 and *Aeromonas veronii*-infected Yangtze finless porpoise: DIS1, DIS2, DIS3, DIS4, DIS5, DIS6, DIS6, DIS7, DIS8) by using sterile tool. All the samples were stored in sterile plastic and transported to the laboratory in ice boxes and then stored at − 80 °C for further study.

### gDNA extraction

Prior to the gDNA extraction, all the samples need to be preprocessed. Initially, the intestinal contents were washed three times with phosphate buffer solution and then the PBS-washed intestinal contents were centrifuged at 500 rpm for 4 min to obtain the sediments. Afterwards, the obtained sediments were resuspended with PBS to further study. The gDNA of each sample was extracted via QIAamp DNA Mini Kit (QIAGEN, Hilden, Germany) according to manufacturer’s recommendations. The extraction quality of gDNA was evaluated by using 0.8% agarose gel electrophoresis. Simultaneously, the Nanodrop™spectrophotometer (NanoDrop Technologies, Thermo Scientific, USA) was used to quantify the concentration of gDNA.

## 16S rRNA gene amplification and sequencing

Universal primers (338F: ACTCCTACGGGAGGCAGCA and 806R: GGACTACHVGGGTWTCTAAT) with barcode were synthesized based on conserved regions in the sequence to amplify the V3–V4 regions of the 16S rRNA. The 2% agarose gel electrophoresis and gel recovery kit (AXYGEN, USA) were used for PCR amplification product evaluation and target segment recovery, respectively. The PCR-recycled products were quantified on Microplate reader (BioTek, FLx800) using Quant-iT PicoGreen dsDNA Assay Kit. The TruSeq Nano DNA LT Library Prep Kit (Illumina, USA) was used to prepare the sequencing library. The sequence ends of the above-mentioned amplified products were repaired by using End Repair Mix2. The self-connected fragments of linker were removed via using magnetic bead screening and the sequencing library with linker was purified. The above DNA fragments with linker were PCR amplified to enrich the sequencing library and BECKMAN AMPure XP Beads was used to purify the library enrichment product. The same volume of 1 × loading buffer (contained SYBR green) was mixed with the PCR products, and detection was performed by using 2% agarose gel electrophoresis on electrophoresis system (DYCZ-20A, Beijing, China). Moreover, The PCR products were mixed in equidensity ratios and purified using GeneJETTM Gel Extraction Kit (Thermo scientific).

Prior to the sequencing, the sequencing libraries were required to be inspected on Agilent Bioanalyzer via using Agilent High Sensitivity DNA Kit. The qualified library has only one peak and no linker. Furthermore, the Quant-iT PicoGreen dsDNA Assay Kit was used to quantify the libraries by the PromegaQuantiFluor fluorescence quantification system and the library concentrations above 2 nM were considered qualified. The qualified libraries were subjected to high-throughput sequencing after gradient dilution and NaOH denaturation into single strands. The raw sequence data has been submitted to the NCBI Sequence Read Archive (SRA) under accession no. PRJNA623474.

### Bioinformatics and statistical analysis

The initial data of high-throughput sequencing was subjected to screening based on the sequence quality via using QIIME software. The sequence was identified and allocated into the corresponding sample based on the primers and barcode information and removed the chimera and interrogative sequences. The obtained sequences with 97% similarity were merged to the same operational taxonomic units (OTUs), and the representative sequences were used for phylogenetic analysis and taxonomic status identification. The representative sequence of each OTU was taxonomically classified based on the Ribosomal Database Project (RDP) database. The diversity of each sample was evaluated based on the abundance distribution of OTU in different samples and the sparse curves were used to assess the depth of sequencing. Four indexes including ACE, Shannon, Simpson and Chao1 were calculated to assess the alpha diversity. In addition, beta diversity analyses (Principal coordinate analysis) was performed to evaluate the similarity of community structure between different samples. GraphPad Prism 7 and R (v3.0.3) software were used for data analysis. Additionally, p-values less than 0.05 were considered statistically significant and the values were presented as mean ± SD.

## Results

### Sequences analyses

**Fig. 1:**
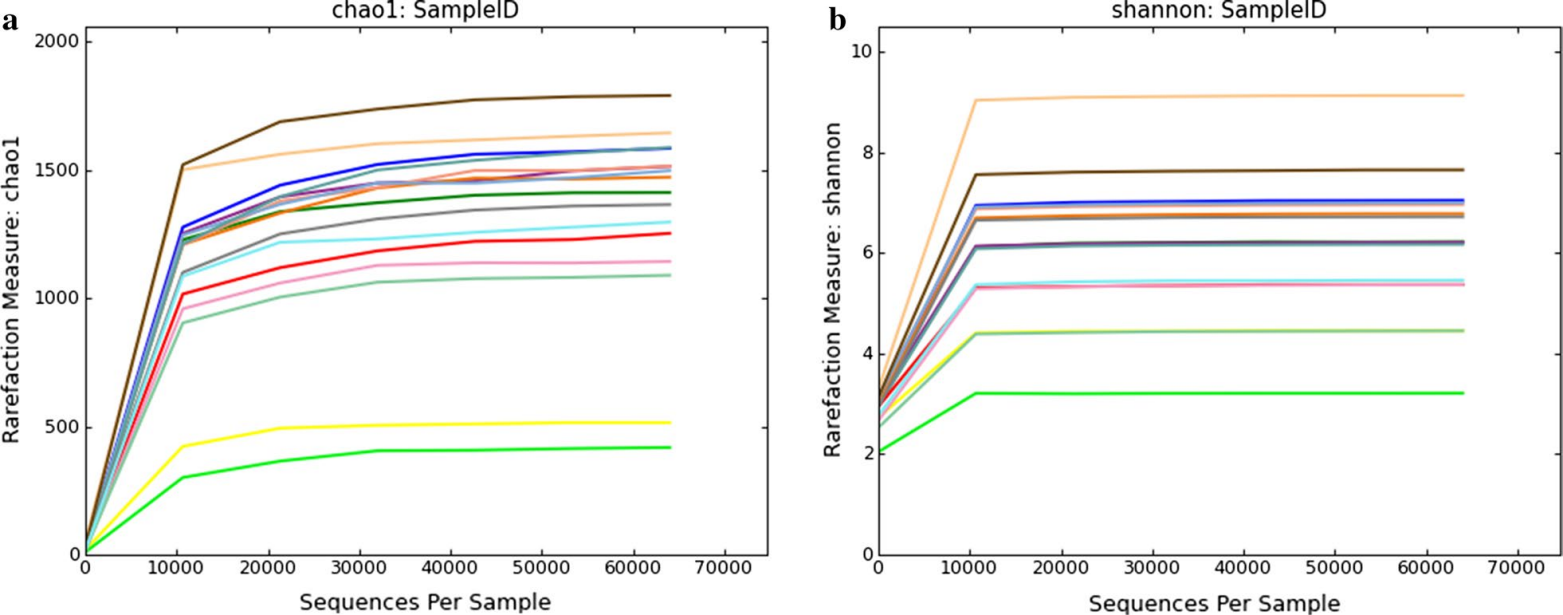
Feasibility analysis of different samples. Each curve indicates a sample. The rarefaction curves (A, B) were used to assess the adequacy of sequencing for each sample. Rank abundance curve (C) was used to evaluate the evenness and abundance of samples. CK indicates the healthy Yangtze finless porpoise, while DIS represents the Yangtze finless porpoise infected by *Aeromonas veronii*

**Fig. 2:**
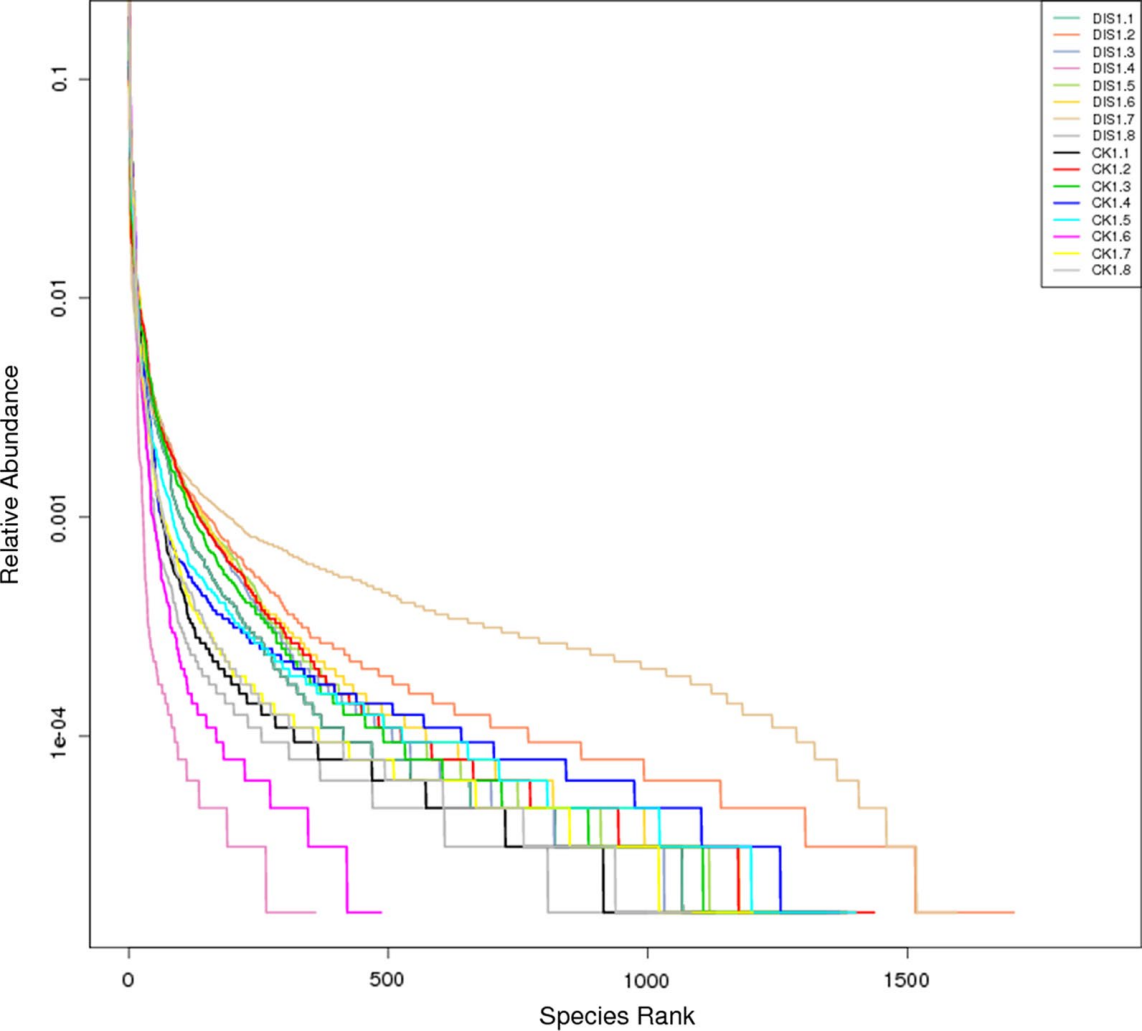
The rank abundance curve of different samples. The rank abundance curve was used to evaluate the evenness and abundance of samples

**Fig. 3:**
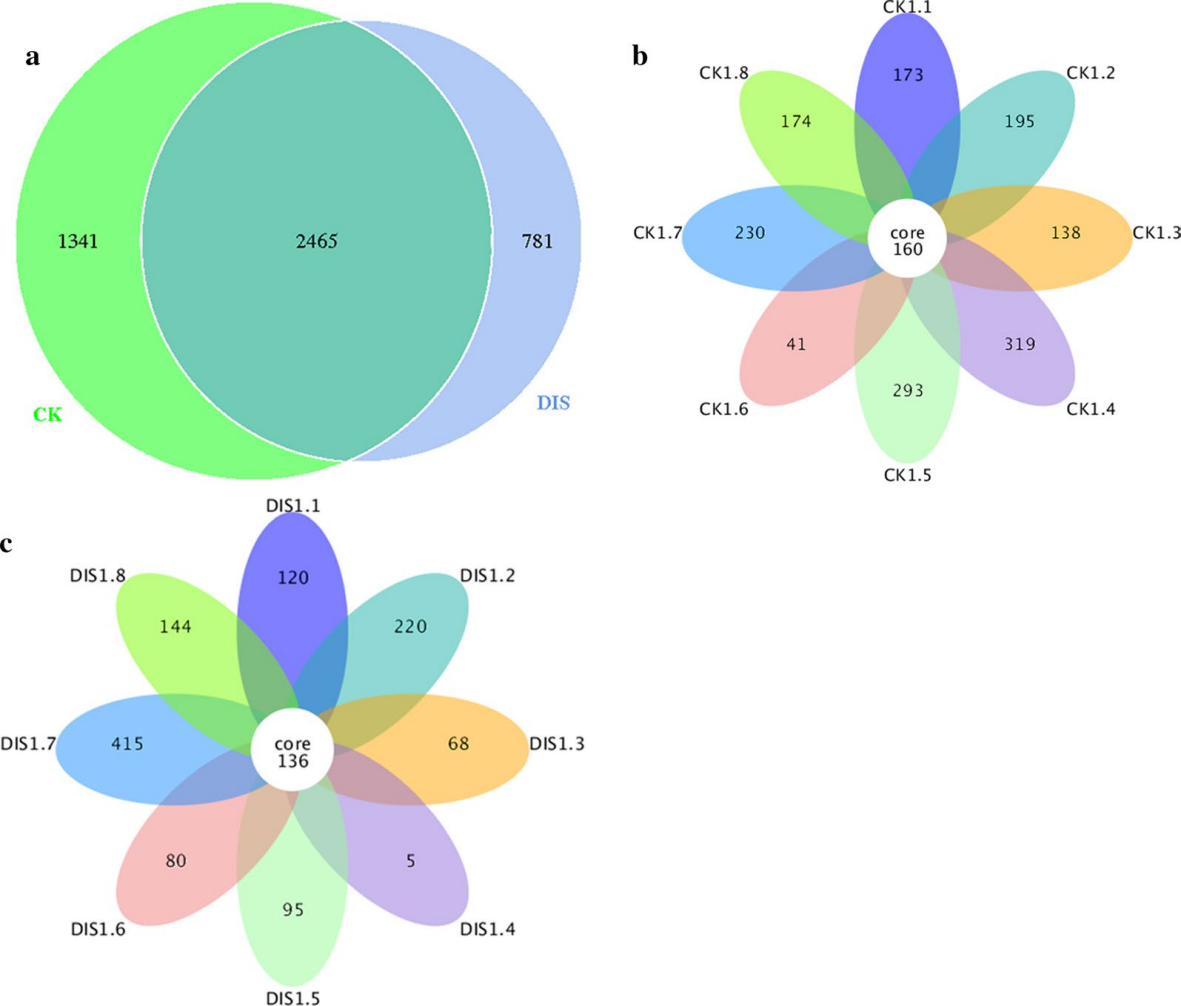
Venn diagrams of the OTUs composition. **a** Venn diagrams of comparison in CK and DIS groups. **b** Venn diagrams for core OTUs composition in the CK group. **c** Venn diagrams for core OTUs composition in the DIS group. CK indicates the healthy Yangtze finless porpoise, while DIS represents the Yangtze finless porpoise infected by *Aeromonas veronii*

It is well known that a number of erroneous or questionable sequences can be produced during the high throughput sequencing. Therefore, the effective sequences were further evaluated and filtered to obtain reliable sequences which can be used for subsequent analysis. The length of qualified sequence should be greater than 150 bp and ambiguous base N was also not allowed. Moreover, the sequences with > 1 mismatched bases at 5′ end or contained > 8 same bases in succession were discarded. In the present study, a total of 127,3276 high-quality sequences were obtained from 16 samples. Additionally, the number of valid sequences from CK group ranged from 69,077 to 85,150, while the number of valid sequences from DIS group ranged from 80,055 to 80,270 (Table 2). The Chao1 and Shannon curves in each sample were extended all the way to the right end of the x-axis and the Rank abundance curve exhibited high and long slight degree broken line. The results of rarefaction curves (Chao1 curve and Shannon curve) and rank abundance curve suggested that the total number of sequences, depth, abundance and evenness meet the requirement for sequencing and analysis (Figs. 1, 2). The OTUs were recognized on the basis of 97% nucleotide-sequence similarity. Therefore, a total of 3806 and 3246 OTUs were identified in the CK and DIS groups, respectively, and 2465 OTUs in common (Fig. 3a). Meanwhile, 160 and 136 core OTUs were found in the CK and DIS groups, respectively (Fig. 3b, c). Additional file 1: Table S1 showing the number of OTUs identified for each sample at different levels.

**Table 2:** The sequence information of each sample

Sample	Raw_reads	Clean_Reads	Effective (%)
CK1.1	88,495	80,121	90.54
CK1.2	83,858	80,213	95.65
CK1.3	87,111	80,281	92.16
CK1.4	83,036	80,061	96.42
CK1.5	74,023	69,077	93.32
CK1.6	82,390	80,182	97.32
CK1.7	90,020	85,150	94.59
CK1.8	81,767	76,856	93.99
DIS1.1	82,345	80,179	97.37
DIS1.2	87,301	80,270	91.95
DIS1.3	85,890	80,168	93.34
DIS1.4	84,149	80,055	95.13
DIS1.5	88,427	80,149	90.64
DIS1.6	81,832	80,134	97.93
DIS1.7	88,893	80,148	90.16
DIS1.8	86,681	80,232	92.56

CK: the healthy Yangtze finless porpoise; DIS: the Yangtze finless porpoise infected by *Aeromonas veronii*

### Analysis of microbial community diversity in different groups

**Fig. 4:**
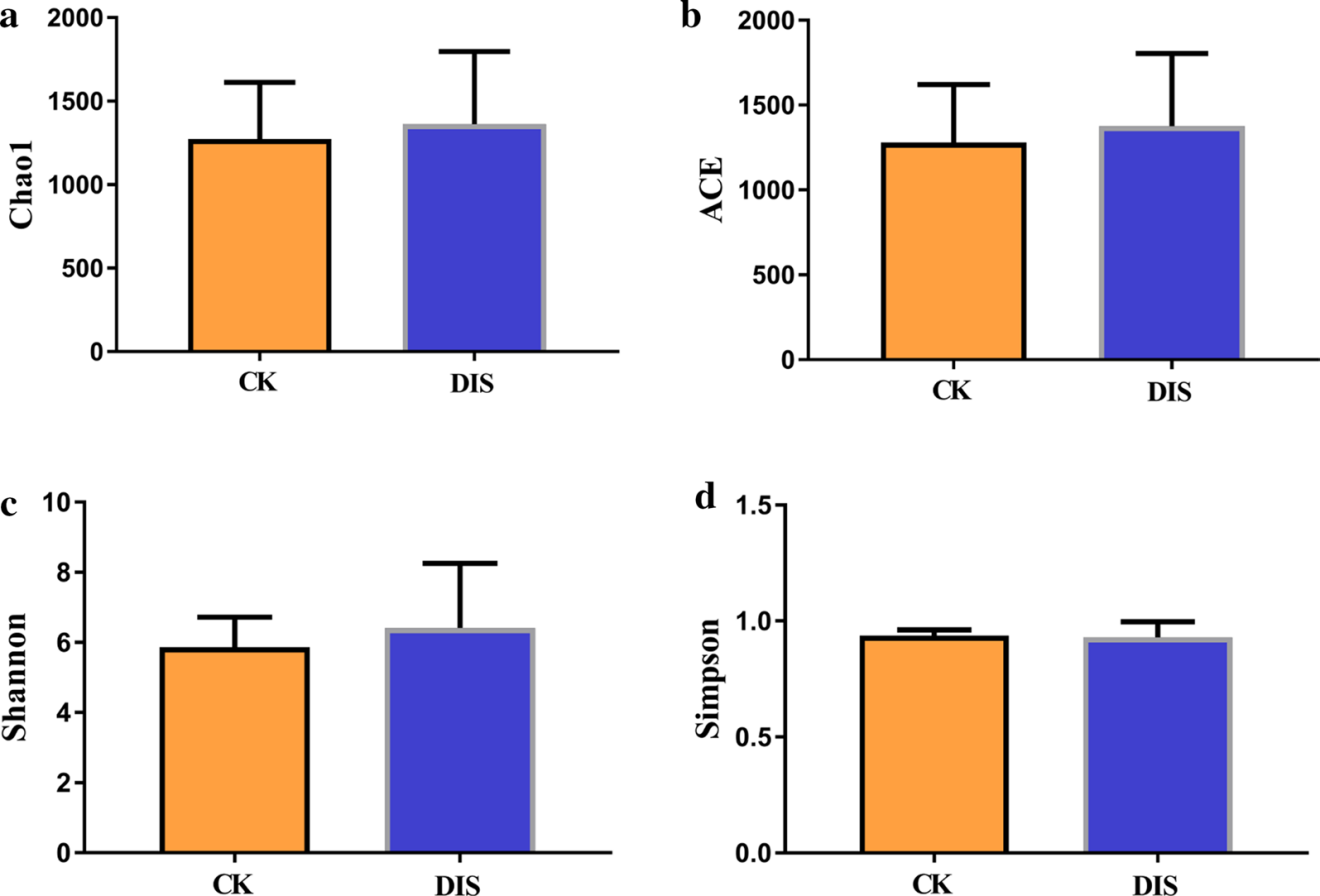
The diversity indices of intestinal microbial community in different groups. The alpha diversity of intestinal flora can be reflected by the Chao1 (**a**), ACE (**b**), Shannon (**c**) and Simpson (**d**). CK and DIS indicate the healthy and *Aeromonas veronii*-infected Yangtze finless porpoise, respectively

**Fig. 5:**
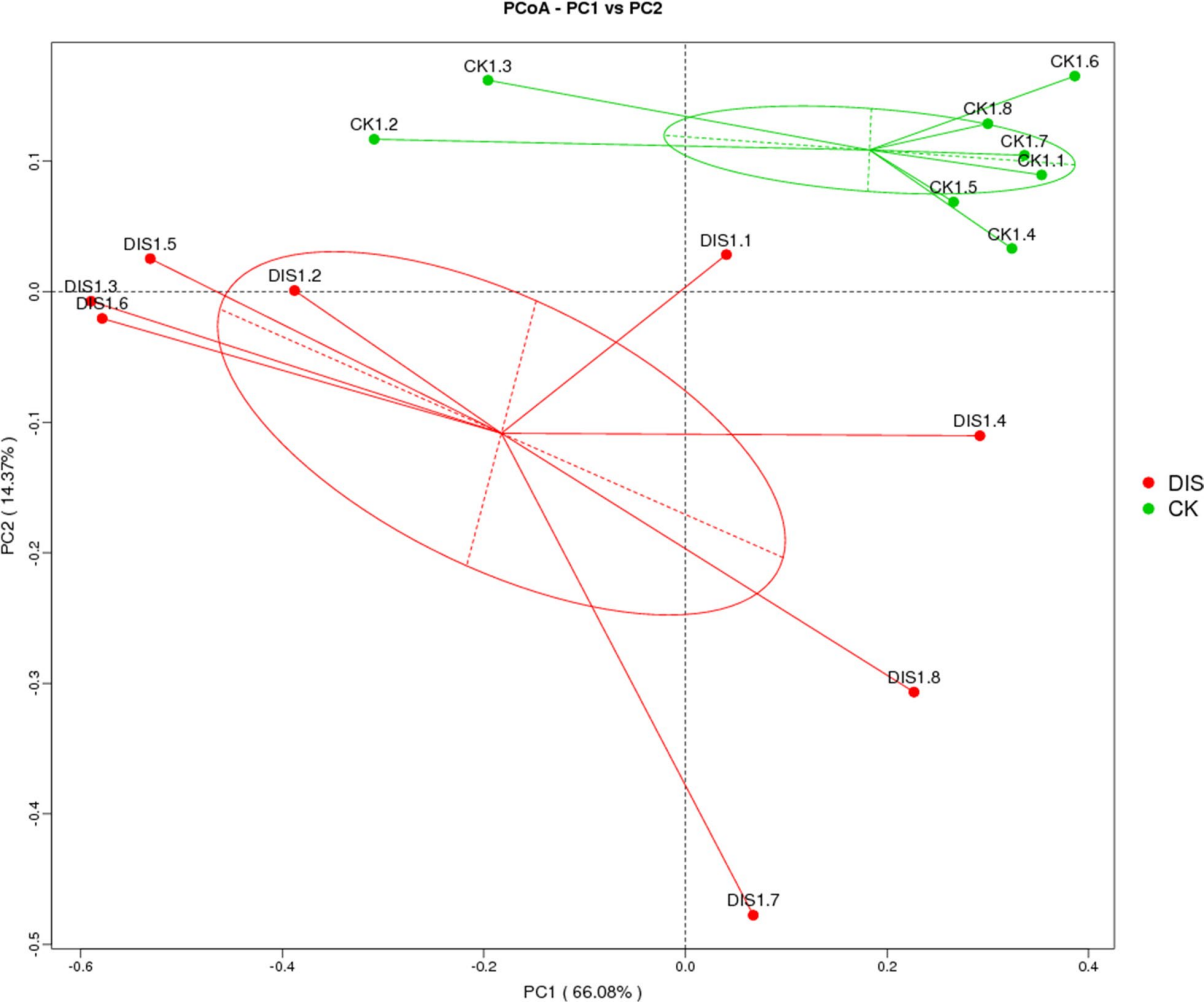
Principal coordinate analysis (PCoA) of intestinal flora. Each dot indicates one sample. The distance of the two points indicates the difference of gut microbiota. CK and DIS refer to healthy and *Aeromonas*-infected Yangtze finless porpoise, respectively

In the current study, Chao1, ACE, Shannon and Simpson were used to evaluate the alpha diversity of the intestinal microbial community (Table 3). The average of Chao1 and ACE indices in DIS group (1362.56 and 1376.007) were higher than those of CK group (1272.937 and 1278.566), while no significant difference was observed between the two groups (P > 0.05) (Fig. 4a, b). The results of Chao 1 and ACE indices suggested that there was no significant difference in the intestinal flora abundance between the two groups. The average of Shannon index was 5.861 and 6.41 in CK group and DIS group, respectively, with no significant difference between two groups (P > 0.05) (Fig. 4c). The average of Simpson index was 0.937 in CK group, 0.929 in DIS group, with no obvious difference in the two groups (P > 0.05) (Fig. 4d). The Shannon and Simpson indices revealed that the difference in the flora evenness between CK and DIS group was non-significant. The PCoA analysis clearly showed the difference among all sample individuals or groups. The results demonstrated that all the samples clustered closely into the two categories, whereas the DIS1.4 and DIS1.8 were specific (Fig. 5).

**Table 3:** The diversity indices of gut microbiota in CK and DIS groups

Sample	Chao1	ACE	Shannon	Simpson
CK1.1	1586.6	1605.92	6.168	0.944
CK1.2	1789.3	1791.01	7.65	0.974
CK1.3	1364.1	1402.71	6.718	0.952
CK1.4	417.75	437.942	3.211	0.799
CK1.5	1514.03	1536.16	6.959	0.963
CK1.6	1496.38	1497.5	6.995	0.958
CK1.7	1643.59	1625.41	9.128	0.994
CK1.8	1088.73	1111.43	4.45	0.85
DIS1.1	1252.21	1254.47	5.37	0.927
DIS1.2	1583.38	1605.53	7.042	0.97
DIS1.3	1471.04	1495.17	6.777	0.967
DIS1.4	1411.1	1411.24	6.221	0.948
DIS1.5	1512.92	1505.77	6.209	0.947
DIS1.6	514.224	518.079	4.452	0.899
DIS1.7	1296.16	1286.998	5.457	0.921
DIS1.8	1142.47	1151.28	5.364	0.919

CK and DIS indicate the healthy and *Aeromonas veronii*-infected Yangtze finless porpoise, respectively

### Composition analysis of the microbial community structure in different groups

**Fig. 6:**
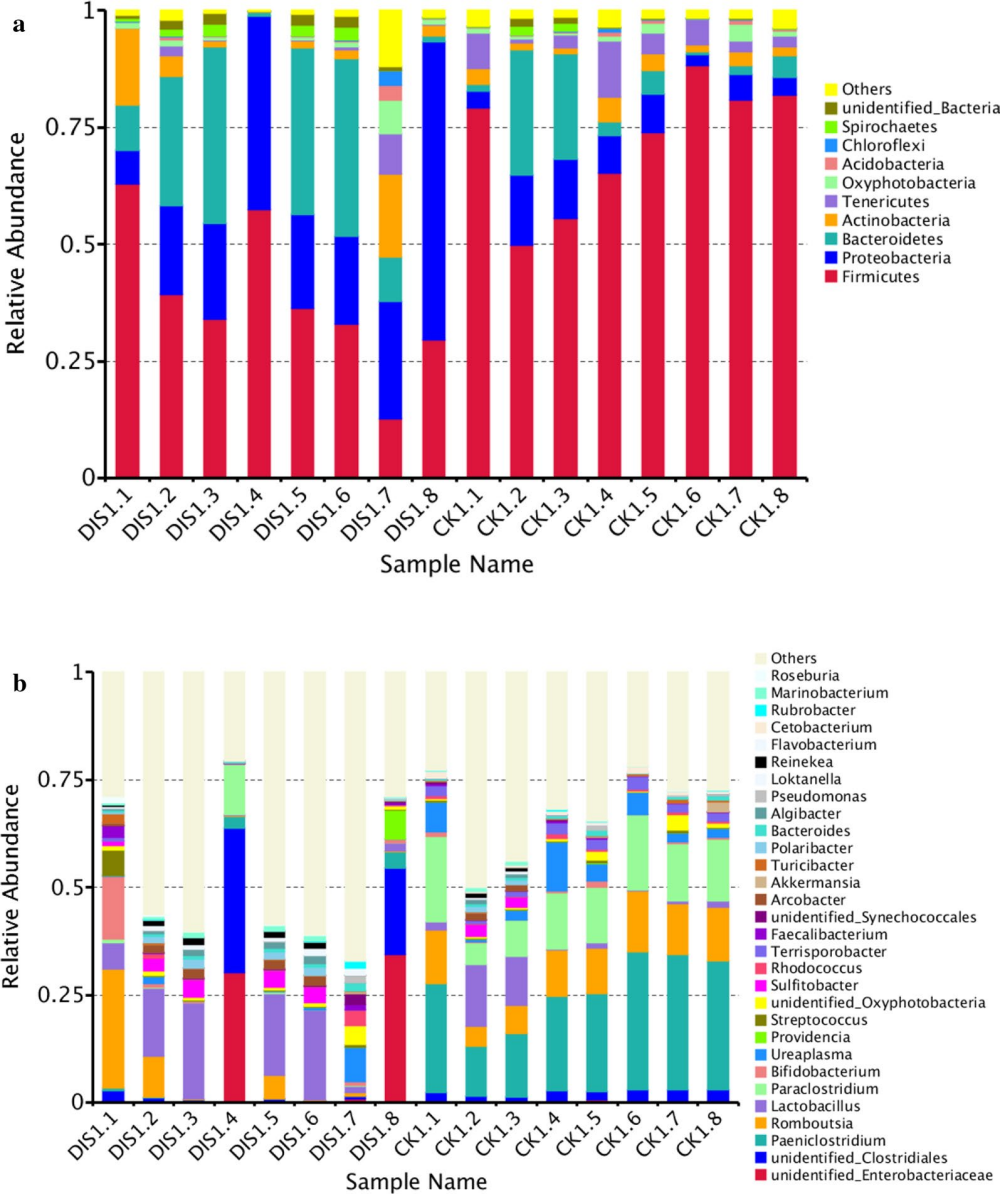
The relative abundance of microbial composition of different samples. **a** The top 10 dominant phylum of the Yangtze finless porpoise intestinal flora. **b** The top 30 primary genera of the Yangtze finless porpoise intestinal flora. CK and DIS refer to healthy and *Aeromonas veronii*-infected Yangtze finless porpoise, respectively

The intestinal microbial community structure in disease and healthy Yangtze finless porpoises were evaluated at different levels, respectively. In the CK group, the preponderant bacteria at phylum level were *Firmicutes* (71.9%), *Proteobacteria* (7.4%), *Bacteroidetes* (8.2%) and *Tenericutes* (4.7%) and the sum of abundance was over 92% (Fig. 6a). Other phyla including *Oxyphotobacteria* (1.2%), *Acidobacteria* (0.4%), *Chloroflexi* (0.2%) and *Spirochaetes* (0.4%) were represented with a lower abundance and the total abundance was less than 3% (Fig. 6a). In the DIS group, *Firmicutes* (38.1%), *Proteobacteria* (27.0%), *Bacteroidetes* (20.0%) and *Actinobacteria* (5.7%) were the four most dominant phyla with a little difference from the control group (Fig. 6a). At the level of genus, the top 30 dominant genera in all collected samples are shown in Fig. 6b. Specifically, *Paeniclostridium* (23.7%), *Romboutsia* (10.4%), *Lactobacillus* (3.9%) and *Paraclostridium* (13.1%) were the predominant bacteria genera in the CK group. Meanwhile, *Lactobacillus* (11.0%), *unidentified_Clostridiales* (7.4%), *unidentified_Enterobacteriaceae* (8.3%) and *Romboutsia* (5.5%) were observed as the predominant in the DIS group. Remarkably, *unidentified_Clostridiales* (33.7% and 20.0%) and *unidentified_Enterobacteriaceae* (30.1% and 34.4%) were the most predominant genera in the DIS1.4 and DIS1.8, respectively.

**Fig. 7:**
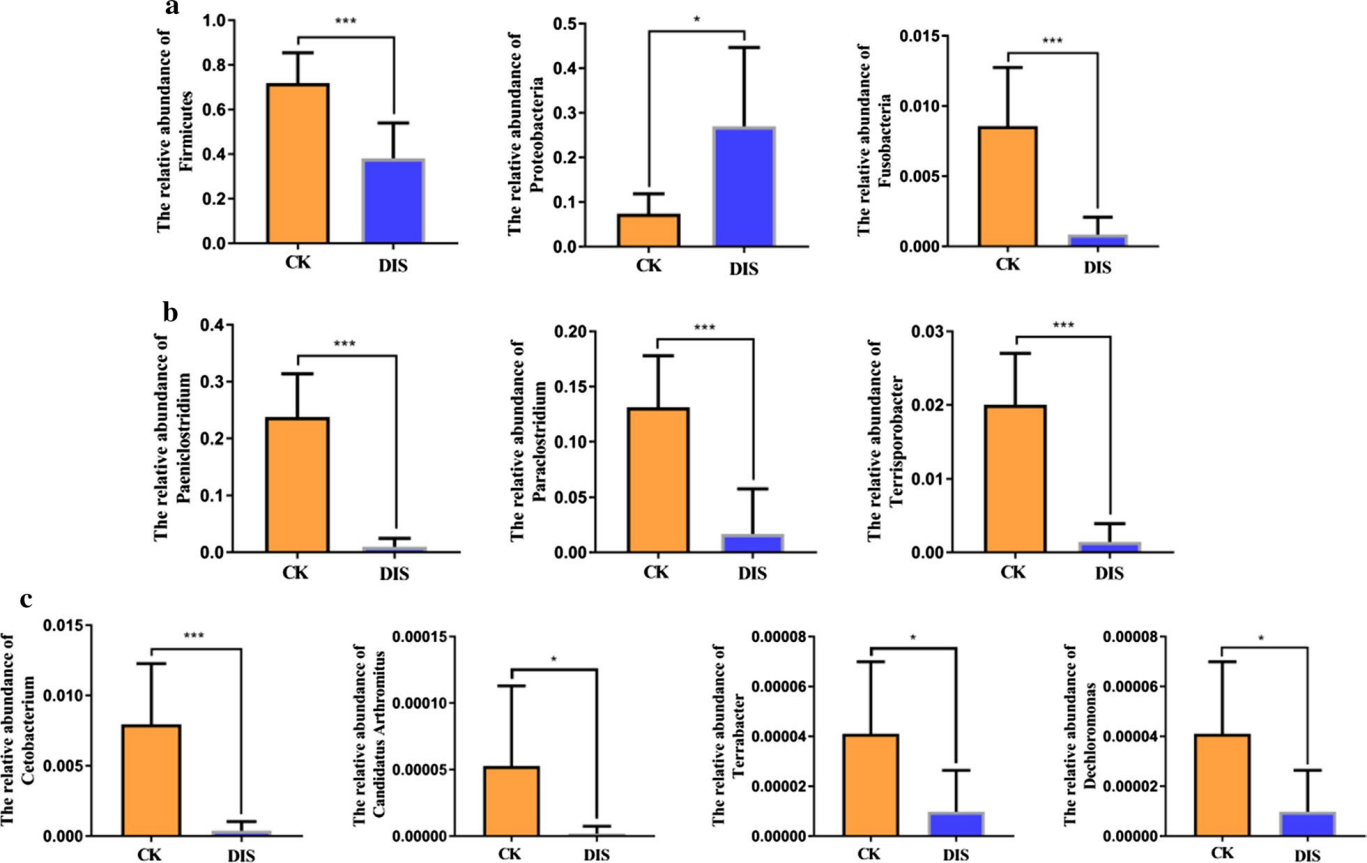
Differences in intestinal bacteria abundance between the CK and DIS groups. **a** Differences in phylum abundance between the CK and DIS groups. **b**, **c** Differences in genus abundance between the CK and DIS groups. CK: the healthy Yangtze finless porpoise. DIS: the Yangtze finless porpoise infected by *Aeromonas veronii*. The results were evaluated through one-way ANOVA. All of the data represent mean ± SD. *P < 0.05, **P < 0.01, ***P < 0.001

The comparison of intestinal bacterial communities between CK and DIS groups were also performed at the levels of phylum and genus. The results suggested that at the phylum level the abundance of *Firmicutes* (P < 0.001) and *Fusobacteria* (P < 0.001) in the CK groups were significantly higher than the DIS group, while the *Proteobacteria* (P < 0.05) content was lower (Fig. 7a). As for genus level, *Paeniclostridium* (P < 0.001), *Paraclostridium* (P < 0.001), *Terrisporobacter* (P < 0.001), *Cetobacterium* (P < 0.001), *Candidatus Arthromitus* (P < 0.05), *Terrabacter* (P < 0.05) and *Dechloromonas* (P < 0.05) were more preponderant in the CK group than the DIS group (Fig. 7b, c).

## Discussion

It is well known that the gut microbial community is an interactive and complex system which has a great influence on the host. Intestinal microbial community is an important barrier for the organism to resist the invasion and colonization of foreign pathogens that plays a key role in the prevention and treatment of diseases. Therefore, it is meaningful to conduct studies on the composition of intestinal flora in different species. Up till now, a large amount of studies has been performed to investigate the role of intestinal flora in a wide range of dysfunctions and diseases [25, 26]. With rapid advancement and development of high-throughput sequencing technology, the composition of gut microbiota community has been investigated in many species, including chicken, camel, piglet, grass carp and cattle [27–29]. However, few studies have focused on the relationship between microbial community structure and disease of Yangtze finless porpoise induced by *Aeromonas veronii* [5]. In the current study, we made a comparison of intestinal flora communities between healthy and *Aeromonas veronii*-infected Yangtze finless porpoise by using high-throughput sequencing.

Considering the scarcity of the species, we selected fecal samples as the research object to evaluate the diversity of intestinal microorganisms. Our results suggested that the number of OTU in the healthy Yangtze finless porpoise was higher than that in the *Aeromonas veronii*-infected Yangtze finless porpoise. However, the results of Chao1, ACE, Shannon and Simpson showed that there was no obvious difference in the abundance and evenness of microbial diversity in Yangtze finless porpoise of different groups, which was consistent with previous studies in the zebrafish with intestinal flora imbalance [30].

The interaction of microbe in the intestines can lead to a huge influence on the immunity, nutrition and health of the organism [31]. It is well-known that *Firmicutes*, *Proteobacteria* and *Bacteroidetes* are the most dominant phyla of the mammals and the percentage of each phylum can be affected by animals’ species, environment, feed [32]. In the current study, the dominant phyla were *Firmicutes*, *Proteobacteria* and *Bacteroidetes* in all the samples, which was in line with many studies in intestinal flora of mammals [33–35]. However, there were significant differences in *Firmicutes* and *Proteobacteria* between the CK and DIS groups. *Proteobacteria* is the largest phylum, which contains a large number of gram-negative pathogenic bacteria, such as *Vibrio cholerae*, *Salmonella* spp., *Helicobacter Pylori* and *Escherichia coli* [36, 37]. The higher abundance of *Proteobacteria* in the intestines increase the risk of pathogen infection. *Firmicutes* are mainly composed of some gram-positive bacteria including *Lactobacillus* spp., *Listeria* spp. and *Lactococcus* spp. [38]. Some studies have shown that *Firmicutes* were closely related to carbohydrate and protein digestion [39–41]. The high abundance of *Firmicutes* in the intestine will contribute to meet the nutritional and energy requirements of host during growth and development [39, 40]. *Lactobacillus* spp., *Listeria* spp. and *Lactococcus* spp. are considered probiotics and play an important role in resisting pathogenic bacteria, maintaining intestinal flora balance and improving immunity [42, 43]. Compared with the CK group, the DIS group had lower *Firmicutes* content and higher *Proteobacteria* level, which indicated that the intestinal flora of the *Aeromonas veronii*-infected Yangtze finless porpoise was imbalanced. Generally, the composition of gut microbiota will change with the influence of external environment to some extent [44]. Although, the intestinal microbial community is in dynamic changes, it may still maintain its functional stability due to the existence of a large number of functionally redundant species [45]. However, significant changes in microbial community will cause intestinal flora imbalance and affect its function. Previous studies have suggested that the observable changes in intestinal flora of young aquatic animals may be one of the reasons for the high mortality [46]. Furthermore, fish with intestinal flora alternation are more susceptible to invasion by pathogenic microorganisms [47, 48].

*Cetobacterium* spp. mostly inhabits in the intestines of fish and contribute to fermentation of peptides and carbohydrate [49, 50]. Additionally, *Cetobacterium* spp. has already been reported to produce vitamin B12 and was considered an important symbiotic organism that provides vitamin B12 to the host [51, 52]. Vitamin B12 can only be synthesized by microorganisms in the host. Vitamin B12 can promote the development and maturation of red blood cells, prevent pernicious anemia and maintain the health of the nervous system. Prior research has shown that the abundance of genus *Cetobacterium* was significantly decreased in graphene-induced intestinal microflora imbalance in zebrafish and cadmium-induced intestinal flora alteration in carassius auratus gibelio [53, 54]. Ma et al. indicated that the level of *Cetobacterium* was significantly decreased in Yunlong Grouper with intestinal microbiota alteration [55]. Moreover, Parshukov et al. showed that the relative abundance of *Cetobacterium* in bacteria infected *Oncorhynchus mykiss* was significantly lower than in healthy *Oncorhynchus mykiss* [56]. In the present study, the abundance of *Cetobacterium* in the DIS group was significantly reduced, which is in accordance with the previous studies of intestinal flora imbalance [53, 54]. *Candidatus Arthromitus* spp., as a gut symbiotic bacterium, plays a vital role in the maturation of the host’s immune system [57]. The lower abundance of genus *Candidatus Arthromitus* in the DIS group, which suggested that the immune system in the *Aeromonas veronii*-infected Yangtze finless porpoise may be affected. In addition, we also found some bacteria related to degradation of pollutants in the CK group. *Terrabacter* spp. is well-known for its versatility in degrading many typical persistent organic pollutants [58, 59]. *Dechloromonas* spp. has reducing capacity and can reduce metal contaminants in water [60, 61]. Therefore, *Dechloromonas* spp. can be used to alleviate water pollution caused by a variety of metal compounds. We speculate that genera *Terrabacter* and *Dechloromonas* in the intestines may contribute to mitigation the toxic effects of pollutants in water. Remarkably, the relative abundance of genera *Enterobacter* and *Clostridium* in the DIS1.4 and DIS1.8 were higher than those in the other samples. *Clostridium* spp. was closely related to intestinal toxemia and diarrhea in mammals and its toxins affect the host health by different pathways [62]. Additionally, *Clostridium* spp. has also been shown to play an important role in causing necrotizing enterocolitis in preterm infants [63]. It has been reported that *Enterobacter* spp. contributed to the occurrence of bacteremia [64]. Therefore, the higher abundance of genera *Enterobacter* and *Clostridium* in the gut may increase the risk of intestinal toxemia, diarrhea and bacteremia.

In conclusion, the current study has compared the differences of diversity and composition about gut flora structure between healthy and *Aeromonas veronii*-infected Yangtze finless porpoise for the first time. The results suggested that there was no significant difference in abundance of intestinal flora between the two groups. The abundance of *Firmicutes* and *Fusobacteria* in the Yangtze finless porpoise were significantly decreased, while, the abundance of *Proteobacteria* was significantly increased after *Aeromonas veronii* infection. Furthermore, *Aeromonas veronii* altered the primary intestinal flora composition in Yangtze finless porpoise by decreasing the relative abundance of genera *Paeniclostridium*, *Paraclostridium*, *Terrisporobacter*, *Cetobacterium* and *Candidatus Arthromitus*. Importantly, we also observed that the levels of genera *Terrabacter* and *Dechloromonas* were reduced in the *Aeromonas veronii*-infected Yangtze finless porpoise, which can alleviate the toxic effects of pollutants in water. However, the gut microbiota can be affected by external environment (water quality and temperature), host diet, host requirement and age. Therefore, we cannot eliminate all the influential elements due to sample size and experiment conditions. However, our study indicated that *Aeromonas veronii* infection caused alterations in the gut microbiota of the Yangtze finless porpoise. This provides a new consideration for the prevention and treatment of Yangtze finless porpoise disease.

## Supplementary information

**Additional file 1: Table S1. The OTUs classification of all samples at different levels.**

## Data Availability

Yes.
